# A Successful View Day

**Published:** 1920-07-10

**Authors:** 


### A Successful View Day.

ON Thursday afternoon, July 1, the Great Northern Central Hospital, in Holloway Road, celebrated one of the pleasantest View Days in the whole of its history, and this notwithstanding the depressing character of the weather.

After the tour of inspection the Chairman made a speech to the visitors when they assembled in the Board Room. He explained in detail the necessity for at least £57,000 for the hospital, as contrasted with the £1,600 which is assured by the endowment. He explained the working branches of the hospital and the usual charges for patients, though in each case absolutely necessitous patients are treated gratuitously. They could be summarised as follows:—

General Wards: From a few shillings per week to 21s.

Cubicle Wards: £1 1s. to £4 4s. per week; and another grade about to be provided—*i.e.*, private wards, with charges from £4 4s. to £6 6s. per week.

Out-patients: 1s. to 5s., according to the dressings or operation required.

He pointed out that his out-patients' department, when built, was intended to provide for 40,000 annual attendances. The attendances at present are at the rate of 200,000 per annum. The casualty department, again, when erected, served a largely residential area. This now is more commercial, and consequently provides many more casualties. A hundred cases a day was not uncommon, instead of the usual twenty per day at the beginning.

Including the Convalescent Home at Clacton, the hospital had 310 beds, but there was at the present moment no fewer than 300 urgent cases on the waiting-list. Before any extension in beds was possible it was absolutely essential to increase the income. It was hoped, partly by the development of a branch for private wards, to obtain from private patients' payments, or payments made on behalf of patients, about £20,000 of the minimum of £57,000 required this year. They were now appealing for this balance of £37,000 for the treatment of necessitous cases.

A certain amount of criticism had been directed against the hospital for endeavouring to extend its work while there was a shortage of funds. But the extensions needed would result in very little increase in expenditure for maintenance, and might result in a positive saving. An instance of this was afforded by the nurses' quarters, which at present were six old, dilapidated houses, whereas it was desired to house them in one central building in Manor Gardens, the site of which was already secured. The quarters would be more commodious and would be rent free.

The needs of the hospital could be summarised as: £10,000 immediately for maintenance; an additional £4,000 before the end of the year; £15,000 to pay off debt; £3,000 for structural repairs which have been held up by the war.

Money was needed also for extension. Sites were secured, but funds must be provided for the building operations. Among the efforts continuously being made to raise funds might be included a large bazaar and a house-to-house collection in North London twice yearly, the first in November or December next. Special public meetings to explain the work and the necessities of the hospital will also be held from time to time. His Lordship expressed the hope that all those present could organise concerts, fêtes, and garden-parties during the next few months.

During the service of tea to the guests some donations of money and many offers of help were received. Such immediate help is the best of all.

